# Common muscle synergies for balance and walking

**DOI:** 10.3389/fncom.2013.00048

**Published:** 2013-05-02

**Authors:** Stacie A. Chvatal, Lena H. Ting

**Affiliations:** The Wallace H. Coulter Department of Biomedical Engineering, Georgia Tech and Emory UniversityAtlanta, GA, USA

**Keywords:** locomotion, posture, muscle synergy, motor control, electromyography

## Abstract

Little is known about the integration of neural mechanisms for balance and locomotion. Muscle synergies have been studied independently in standing balance and walking, but not compared. Here, we hypothesized that reactive balance and walking are mediated by a common set of lower-limb muscle synergies. In humans, we examined muscle activity during multidirectional support-surface perturbations during standing and walking, as well as unperturbed walking at two speeds. We show that most muscle synergies used in perturbations responses during standing were also used in perturbation responses during walking, suggesting common neural mechanisms for reactive balance across different contexts. We also show that most muscle synergies using in reactive balance were also used during unperturbed walking, suggesting that neural circuits mediating locomotion and reactive balance recruit a common set of muscle synergies to achieve task-level goals. Differences in muscle synergies across conditions reflected differences in the biomechanical demands of the tasks. For example, muscle synergies specific to walking perturbations may reflect biomechanical challenges associated with single limb stance, and muscle synergies used during sagittal balance recovery in standing but not walking were consistent with maintaining the different desired center of mass motions in standing vs. walking. Thus, muscle synergies specifying spatial organization of muscle activation patterns may define a repertoire of biomechanical subtasks available to different neural circuits governing walking and reactive balance and may be recruited based on task-level goals. Muscle synergy analysis may aid in dissociating deficits in spatial vs. temporal organization of muscle activity in motor deficits. Muscle synergy analysis may also provide a more generalizable assessment of motor function by identifying whether common modular mechanisms are impaired across the performance of multiple motor tasks.

## Introduction

Humans and animals are able to robustly move over diverse terrains and withstand challenging disturbances to balance during locomotion. Achieving these remarkable behaviors requires precise and dynamic coordination of multiple muscles across the limbs and trunk via hierarchical neural pathways. However, little is known about how the nervous system integrates the concurrent control of locomotion and balance functions over different movement contexts. Neural circuits for locomotion have been identified in the mammalian spinal cord, and can endogenously produce rhythmic motor patterns to muscles (Brown, 1911; Grillner, 1975; Rossignol et al., 1996). These patterns can be modified by sensory feedback (Forssberg et al., 1980; Quevedo et al., 2000; Rossignol and Bouyer, 2004) and motor planning mechanisms (Drew et al., 2002) that alter the gait pattern. Perturbations to walking can elicit long-latency muscle responses (Tang et al., 1998; Chvatal and Ting, 2012) as well as alter the locomotor rhythm during stumbling corrective responses (Pijnappels et al., 2005; van Der Linden et al., 2007). During standing balance control, perturbations evoke coordinated long-latency responses in muscles that help to return the body to postural equilibrium; these require brainstem integration of multisensory cues (Macpherson and Fung, 1999; Deliagina et al., 2008).

Are there common neural mechanisms underlying the control of walking and reactive balance control, and how are these mechanisms integrated during natural movements? Recent research demonstrates the neural control of muscles may be modular, organized in functional groups often referred to as muscle synergies (Tresch et al., 1999; Giszter et al., 2007; Ting and McKay, 2007; Drew et al., 2008; Chiel et al., 2009; Yakovenko et al., 2011). Each muscle synergy is proposed to specify a fixed pattern of co-activation across multiple muscles at any given time point. Muscle synergies have been used to describe muscle coordination during a variety of motor behaviors including balance control (Torres-Oviedo and Ting, 2007), walking (Ivanenko et al., 2004; Clark et al., 2010; Chvatal and Ting, 2012), reaching (D'Avella et al., 2006; Muceli et al., 2010), and grasping (Hamed et al., 2007; Acharya et al., 2008; Overduin et al., 2008). Moreover, common muscle synergies have been identified across different motor behaviors such as frog swimming, kicking, and jumping (Hart and Giszter, 2004; Cheung et al., 2005; D'Avella and Bizzi, 2005; Cheung et al., 2009), forward and backward locomotion (Raasch and Zajac, 1999; Ting et al., 1999), and across reactive balance conditions (Torres-Oviedo and Ting, 2010; Chvatal et al., 2011). Based on these findings, we hypothesize that a common set of muscle synergies may be recruited by parallel neural pathways governing voluntary, reactive, and automatic motor behaviors in the upper and lower limbs. Muscle synergy analysis may thus provide a more generalizable assessment of motor function in neuromotor deficits, providing more specific information about functional deficits that may guide more targeted rehabilitation interventions.

It has been demonstrated that the phasic recruitment of muscle synergies underlies variability in locomotor behaviors such as pedaling and walking (Ting et al., 1999; Ivanenko et al., 2004; Krouchev et al., 2006; Clark et al., 2010; Lacquaniti et al., 2012). During locomotion, specific muscle synergies have been associated with a particular phase of the gait cycle (Ivanenko et al., 2004; Krouchev et al., 2006; Clark et al., 2010), despite differences in how muscle synergies are defined (Safavynia and Ting, 2012). Furthermore, the order of recruitment has been shown to be consistent across conditions, such as when subjects concurrently perform voluntary tasks (Ivanenko et al., 2005) or shift from walking to running (Cappellini et al., 2006). Moreover, recruitment of muscle synergies within a particular phase of gait has been shown to be modulated systematically as a function of walking speed, and also to account for cycle-by-cycle variability in the locomotor pattern (Clark et al., 2010). These variations in muscle synergy recruitment may reflect changing task demands across gait conditions. Muscle synergies may be organized to produce specific whole-limb or whole-body biomechanical functions during locomotion (Raasch and Zajac, 1999; Neptune et al., 2009; Allen and Neptune, 2012) such that altering the phase, amplitude, or duration of muscle synergy recruitment may produce a variety of locomotor behaviors (Raasch and Zajac, 1999; Ting et al., 1999; McGowan et al., 2010). This is consistent with the idea that muscle synergies reflect motor modules that allow the nervous system to produce consistent biomechanical functions.

Rapid and complex changes in the coordination of muscles are required to recover from discrete perturbations that produce large disruptions to the locomotor pattern. In response to unexpected obstacles, slipping, waist pulls, or surface heights, immediate and delayed changes to muscle activity and kinematics have been observed (Tang et al., 1998; You et al., 2001; Ferber et al., 2002; Misiaszek, 2003; Oddsson et al., 2004; Chambers and Cham, 2007; van Der Linden et al., 2007; Bachmann et al., 2008; Shinya and Oda, 2010). Perturbations typically alter the duration of stance and swing phase such that changes in stance duration, step length, and step width are observed within the perturbed step (Oddsson et al., 2004) and can continue in subsequent steps (Patla, 2003). Corrective muscular responses at long latencies (~100 ms) following perturbations are observed in both the stance and swing limb (Tang et al., 1998; Bachmann et al., 2008) and appear to be superimposed upon the locomotor pattern (Gorassini et al., 1994; Hiebert et al., 1994). Moreover, different motor strategies to maintain whole-body stability can be evoked, consistent with the idea that long-latency responses are organized to maintain task-level goals (Nashner, 1976; Horak and Macpherson, 1996; Carpenter et al., 1999; Chvatal et al., 2011), and not simply limb posture (cf. autogenic reflex). We recently demonstrated that the same muscle synergies recruited phasically during overground walking were also recruited at long-latencies in response to discrete perturbations to walking (Chvatal and Ting, 2012). However, it remains unknown whether these long-latency responses are similar in organization to those evoked during perturbations to standing balance, which would support the idea of a common mechanism mediating balance responses across movement contexts.

In discrete perturbations to standing balance control, the modulation of a few muscle synergies can robustly explain variations in muscle activity across reactive balance responses during different perturbations to standing. In response to multidirectional support-surface perturbations, muscle synergy recruitment is directionally tuned to perturbation direction, and generates a specific biomechanical function (e.g., ground-reaction force direction) to restore the center-of-mass (CoM) in both humans and animals (Ting and Macpherson, 2005; Torres-Oviedo et al., 2006; Chvatal et al., 2011). Moreover, the multidirectional recruitment of muscle synergies during standing balance can be predicted based on the deviation of CoM kinematics from the upright, static state, such that they reflect the task-relevant error for maintaining postural equilibrium and orientation (Safavynia and Ting, 2013). Variations in muscle activity from trial to trial in identical perturbation conditions (Torres-Oviedo and Ting, 2007; Safavynia and Ting, 2012) as well as across biomechanical contexts [e.g., standing with wide, narrow, crouched, or single-limb stance (Torres-Oviedo et al., 2006; Torres-Oviedo and Ting, 2010)], can be accounted for by the differential modulation of a common set of muscle synergies for balance. Furthermore, muscle activity and the resulting force production during reactive non-stepping and stepping responses were explained by a common set of muscle synergies (Chvatal et al., 2011), demonstrating the robustness of the muscle synergy organization and function in mediating a variety of balance behaviors.

Here, we hypothesized that a common set of lower-limb muscle synergies mediate reactive balance and walking. Recent studies during perturbed locomotion demonstrate that locomotor muscle synergies are recruited at long-latencies following discrete perturbations to walking (Chvatal and Ting, 2012; Oliveira et al., 2012). This suggests that muscle synergies recruited rhythmically for locomotion may also be recruited during atypical phases of gait due to sensorimotor feedback mechanisms governing long-latency balance responses. However, it is not known whether the same muscle synergies are recruited during long-latency balance responses evoked during standing and walking. To compare muscle activity during directional balance control, we imposed twelve directions of support-surface perturbations during standing and walking at self-selected and slow speeds. First, we predicted that reactive balance to multidirectional perturbations across contexts, e.g., during standing and walking, were mediated by common muscle synergies. We then predicted muscle activity in both reactive balance and overground walking were also mediated by common muscle synergies. Our results suggest that a common set of muscle synergies is differentially recruited by neural circuits mediating reactive balance across movement contexts and for locomotion.

## Methods

In order to determine whether common muscle synergies are recruited during postural responses to perturbations in different dynamic contexts, we recorded postural responses to ramp and hold translations of the support surface during standing balance as well as during walking at both self-selected and slow walking speeds. Perturbations in twelve directions in the horizontal plane were delivered in random order in each condition. Muscle synergies were extracted from both the standing balance and walking conditions, as well as from trials of unperturbed walking. Muscle synergies and recruitment coefficients from each condition were compared to give insight into neural mechanisms underlying each condition.

### Data collection

Seven healthy subjects (four male, three female) between the ages of 19 and 26 responded to support surface translations according to an experimental protocol that was approved by the Institutional Review Boards of Georgia Institute of Technology and Emory University. All subjects gave informed consent before participating in each of three experimental blocks (standing balance, self-selected speed walking, and slow walking). The order in which the blocks were presented was randomized for each subject.

**Figure 1:**
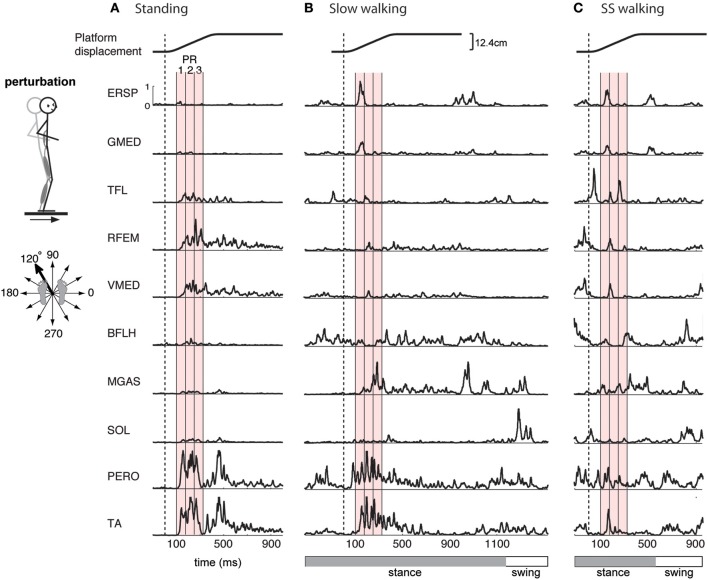
**Examples of EMG activity in perturbation responses during (A) standing, (B) slow walking, and (C) self-selected speed walking.** Responses to a forward and leftward perturbation of the support surface during each condition are shown. Balance perturbations were induced by ramp-and-hold displacement perturbations in 12 evenly spaced directions in the horizontal plane. EMG responses occur 100-ms after the onset of platform motion (vertical dashed line). Shown here are erector spinae (ERSP), gluteus medius (GMED), tensor fascia lata (TFL), rectus femoris (RFEM), vastus medialis (VMED), biceps femoris (BFLH), medial gastrocnemius (MGAS), soleus (SOL), peroneus (PERO), and tibialis anterior (TA) responses. Mean EMG activity was calculated for 3 time bins during the automatic postural response (PR), indicated by the red shaded region, beginning 100 ms (PR1), 175 ms (PR2), and 250 ms (PR3) following perturbation. One complete gait cycle is shown for each walking speed, and the horizontal bar indicates stance (gray) and swing (white) phase. Perturbations during walking were administered in early stance.

In the standing balance block, subjects stood on an instrumented platform that translated in 12 equally spaced directions in the horizontal plane (see Figure 1). Subjects were instructed to maintain balance without stepping if possible. The platform's displacement was 12.4 cm, velocity was 35 cm/s, and acceleration was 0.5 g. Five trials of each of the 12 directions of perturbation were collected in random order. All subjects were able to maintain balance without taking a step.

In the walking blocks, subjects walked overground slowly (0.6–0.7 m/s) or at a self-selected pace (1.2–1.5 m/s) for approximately 7.5 m, or 7 gait cycles. Subjects were instructed to maintain a pace as closely as possible to a metronome beat. Subjects listened to 4 metronome beats and then began walking at self-selected time after the metronome was silenced. In slow trials the metronome was set at 60 bpm, and in self-selected trials the metronome pace was matched to each subject's preferred pace, determined when they first arrived. Subjects began walking with their right foot, and data collection began on the third step to eliminate any variability associated with gait initiation. Eight trials of unperturbed walking were collected at the beginning of each block, in which the subject knew there would be no perturbation. In the remaining trials, subjects were told that there may or may not be a perturbation. Twelve trials of unperturbed walking were collected randomly in between the perturbation trials in order to capture any anticipatory responses. In perturbed trials, perturbations (displacement 12.4 cm, velocity 40 cm/s, acceleration 0.7 g) were applied as subjects crossed the instrumented platform halfway along the path, during early stance phase of the right leg. The perturbation was applied when the ground reaction force of the right foot reached ~60% of body weight as measured by force plates (AMTI, Watertown, MA) embedded in the platform. Perturbation direction was randomized, and three trials of each direction for each walking speed were collected. Data from the four cardinal directions of perturbations and from unperturbed walking trials were analyzed and published previously (Chvatal and Ting, 2012).

Surface EMG activity was recorded from sixteen muscles of the lower-back and leg on the subject's right side, the stance leg in perturbed walking. Muscles recorded included: vastus lateralis (VLAT), rectus femoris (RFEM), rectus abdominis (REAB), biceps femoris long head (BFLH), semitendinosus (SEMT), adductor magnus (ADMG), erector spinae (ERSP), abdominal external oblique (EXOB), vastus medialis (VMED), tibialis anterior (TA), medial gastrocnemius (MGAS), lateral gastrocnemius (LGAS), soleus (SOL), peroneus (PERO), tensor fasciae latae (TFL), and gluteus medius (GMED). EMG data were sampled at 1080 Hz, high pass filtered at 35 Hz, de-meaned, rectified, and low-pass filtered at 40 Hz, using custom MATLAB routines. Additionally, kinetic data was collected at 1080 Hz from force plates under the feet, and kinematic data was collected at 120 Hz using a motion capture system (Vicon, Centennial, CO) and a custom 25-marker set that included head-arms-trunk (HAT), thigh, shank, and foot segments.

### Data processing

In order to identify muscle synergies, we first generated EMG data matrices from each condition as follows:

In the standing balance condition, three time bins during the automatic postural response were analyzed (Torres-Oviedo and Ting, 2007; Chvatal et al., 2011). The automatic postural response (APR) has been well-characterized and occurs ~100 ms following the perturbation (Horak and Macpherson, 1996). Due to variations in muscle activity during this APR, we further divided it into three 75-ms time bins beginning 100 ms (PR1), 175 ms (PR2), and 250 ms (PR3) after perturbation onset (Figure 1A red shaded areas). Mean muscle activity for each muscle during each time bin was calculated for each trial. These numbers were assembled to form the data matrix used for subsequent muscle synergy analysis, which consisted of 3 time bins × 12 directions × 5 trials = 180 points for each of the 16 each muscles.

Similarly, in the perturbed walking conditions we also analyzed three 75-ms time bins to characterize the reactive response to perturbation. Mean muscle activity was calculated during three time bins beginning 100 ms, 175 ms, and 250 ms after the perturbation (Figure 1B red shaded areas). For perturbed walking, the data matrix consisted of 3 time bins × 12 directions × 3 trials = 108 points for each of the 16 each muscles.

In the unperturbed walking condition, at least three complete gait cycles for each trial were included in the analysis. EMG data were downsampled by averaging the data in 75-ms time bins (Chvatal and Ting, 2012). Reducing the size of the time bins to 10 ms during walking did not affect the number or structure of muscle synergies in prior studies (Chvatal and Ting, 2012), as well as for the current paper (not shown). Time-courses of EMG from unperturbed walking trials of each subject were concatenated to form the data matrix. The size of the data matrix varied across subjects and walking speeds since no time-normalization was performed on walking cycles, but each subject's data matrix had greater than 1044 points for each of the 16 muscles.

For all conditions, the activation of each muscle in each subject was normalized to the maximum activation observed during the unperturbed walking trials at the self-selected walking speed. The elements of each row of a data matrix (each muscle) constructed from unperturbed walking trials at the self-selected speed therefore ranged from 0–1. Identical normalization factors from the unperturbed self-selected walking condition were used for all other conditions for each subject. Tuning curves were generated by plotting the activation of each muscle with respect to perturbation direction within a given time bin.

### Extraction of muscle synergies

We extracted muscle synergies from each data matrix of EMG recordings using non-negative matrix factorization (NNMF) (Lee and Seung, 1999; Tresch et al., 1999), which has previously been used for muscle synergy analysis (Ting and Macpherson, 2005; Torres-Oviedo and Ting, 2007). NNMF assumes that a muscle activation pattern, M, in a given time period is comprised of a linear combination of a few muscle synergies, W_i_ that are each recruited by a synergy recruitment coefficient, c_i_. Therefore, a particular muscle activation pattern, M, would be represented by:

$$\text{M}={\text{c}}_{1}{\text{W}}_{1}+{\text{c}}_{2}{\text{W}}_{2}+{\text{c}}_{3}{\text{W}}_{3}+\ldots$$

where W_i_ specifies the relative contributions of the muscles involved in synergy *i*. Each muscle synergy has a fixed composition, and each is multiplied by a scalar recruitment coefficient, c_i_, which changes over time and across conditions. Prior to extracting muscle synergies, each muscle vector in the data matrix was normalized to have unit variance to ensure equal weighting in the muscle synergy extraction. After extracting muscle synergies, the unit variance scaling was removed from data so that each muscle's data was returned to the scale where 1 is the maximum activation during self-selected speed unperturbed walking, in order to permit comparison of responses and muscle synergies across conditions.

Muscle synergies for reactive balance were identified independently in each of the three perturbation conditions: standing, slow walking, and self-selected walking. We extracted 1–16 muscle synergies, and the goodness of fit of the data reconstruction using each number of muscle synergies was quantified by variance accounted for (VAF), defined as 100 × uncentered Pearson's correlation coefficient (Zar, 1999; Torres-Oviedo et al., 2006). The number of muscle synergies selected to describe each dataset *(Nsyn)* was determined by choosing the least number of synergies that could account for greater than 90% of the overall VAF. We added the further local criterion that muscle synergies also accounted for greater than 75% VAF in each muscle and each perturbation direction. This local fit criterion was more stringent and ensured that relevant features of the data set are reproduced. VAF for each muscle (VAFmus) quantified the extent to which the muscle synergies accounted for variability in the activity of individual muscles across all time bins, perturbation directions, and trials. VAF for each perturbation direction (VAFcond) quantified the extent to which the muscle synergies accounted for the variability in muscle activation patterns formed by the response of all 16 muscles to a single perturbation direction during one time bin across all trials.

To validate the similarity of muscle synergies in reactive balance responses across movement contexts, we further identified muscle synergies using two additional methods that combined perturbation conditions. First, muscle synergies identified from perturbation responses during standing were used to reconstruct the responses during the two walking perturbation conditions. Condition-specific muscle synergies were extracted from walking perturbation response data that was not accounted for by the standing muscle synergies. To this end, we used an iterative algorithm that held fixed the muscle synergies extracted from standing data while optimizing a new set of muscle synergies extracted from the remainder of the variability in the walking perturbation data not accounted for by the standing muscle synergies (Cheung et al., 2009; Torres-Oviedo and Ting, 2010; Chvatal et al., 2011). As a second validation, we extracted muscle synergies from a data matrix containing all three perturbation conditions combined, and compared these to the muscle synergies identified from the independent data sets. In a combined extraction, there is a possibility of one condition dominating the others, so most of the results presented are comparisons of the muscle synergies identified from the independent datasets.

Muscle synergies for walking were first identified from unperturbed walking. Since we previously showed that the similar muscle synergies are identified when each walking speed is analyzed individually (Chvatal and Ting, 2012), one set of muscle synergies was extracted from a data matrix consisting of both self-selected speed unperturbed walking catch trials and slow unperturbed walking catch trials, and these muscle synergies were termed “walking” muscle synergies. For each subject, we selected the least number of muscle synergies (*Nsyn*) that satisfied both the global criterion of reconstructing at least 90% of the overall variance (VAF = 90%) as well as the local criterion of reconstructing at least 75% of the variability in each muscle (Chvatal and Ting, 2012). Once *Nsyn* was selected for each condition, the muscle synergies were used to reconstruct the EMG patterns, and measured and reconstructed data were compared for a particular muscle, time bin, and perturbation direction for each trial to examine the ability of the muscle synergies to account for inter-trial variations. Similarities between measured and reconstructed data were quantified using *r*^2^ and VAF.

We used two methods to validate the similarity of muscle synergies for reactive balance during standing and unperturbed walking. First, muscle synergies identified from perturbation responses during standing were used to reconstruct unperturbed walking data and *vice versa*. Using the algorithm described above, walking-specific muscle synergies were extracted from unperturbed walking data that was not accounted for by the standing muscle synergies, and standing-specific muscle synergies were extracted from standing perturbation response data that was not accounted for by the walking muscle synergies. Significant differences between reconstructions using the various muscle synergy sets were determined using paired *t*-tests. Second, we also extracted muscle synergies from a data matrix containing both unperturbed walking data and standing perturbation response data combined. To ensure an equal amount of walking and standing data in the combined data matrix, only a single trial of unperturbed walking at each walking speed was included. We first verified that the muscle synergies extracted from the single trial of walking at each speed were similar to the walking muscle synergies described above.

### Muscle synergy comparison

To determine similarity in muscle synergies across conditions, we compared muscle synergies extracted from reactive balance during standing and walking, as well as from reactive balance compared to unperturbed walking. When comparing two sets of muscle synergies, we calculated correlation coefficients (*r*) between each muscle synergy vector in the first set and each in the second set. A pair of muscle synergies were considered “similar” if they had *r* > 0.623, which corresponds to the critical value of *r*^2^ for 16 muscles [*r*^2^ = 0.388; *p* = 0.01; see Chvatal et al. (2011) for muscle synergy comparison details].

Across perturbation conditions, we compared the tuning curves of similar synergies to determine if they were recruited for similar perturbation directions in different contexts. We examined the composition and tuning of any condition-specific muscle synergies to determine their potential similarity in function.

## Results

### Differences in individual muscle activation across perturbation conditions

**Figure 2:**
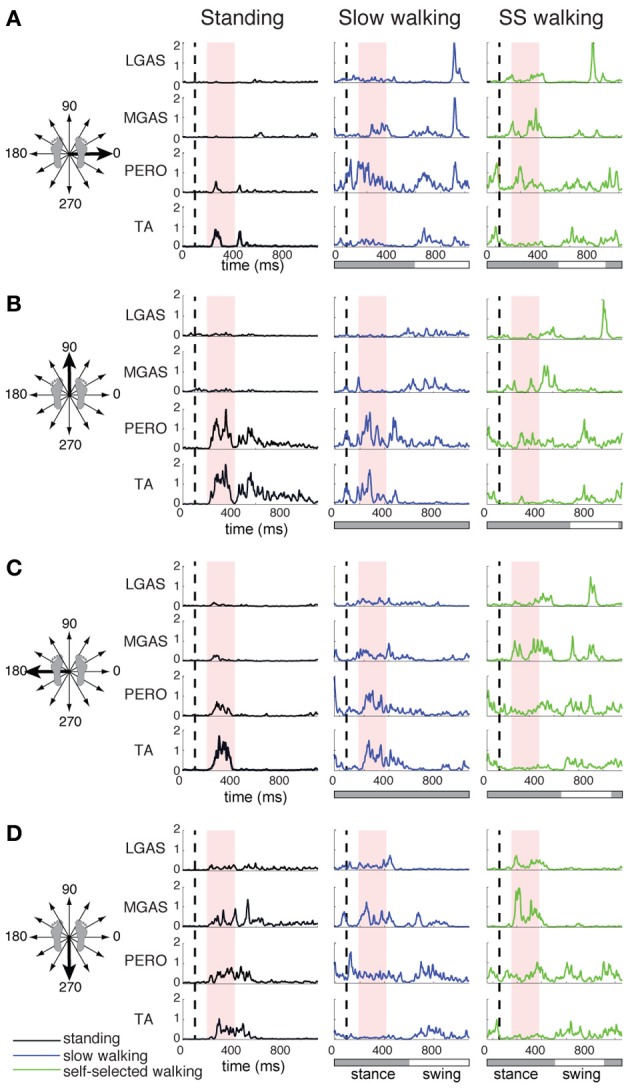
**Lateral gastrocnemius (LGAS), medial gastrocnemius (MGAS), peroneus (PERO), and tibialis anterior (TA) activity during perturbation responses during standing (black), slow walking (green), and self-selected walking (blue), for (A) a rightward perturbation, (B) a forward perturbation, (C) a leftward perturbation, and (D) a backward perturbation.** Vertical dashed line indicates perturbation onset and the red shaded box indicates the perturbation response time window used here for analysis: 100–325 ms after perturbation onset. For walking trials, the horizontal bar indicates stance (gray) and swing (white) phases. Muscle activity was averaged across each of 3 time bins during the postural response time window and plotted against perturbation direction to generate tuning curves shown in Figure 3.

The muscles activated in response to different perturbation directions was generally similar in standing and walking, but could vary greatly in magnitude (Figure 1). For example, in one subject, in response to a forward, rightward perturbation, TA and PERO were strongly activated in standing and slow walking, but more weakly activated in the self-selected walking condition; RFEM was activated strongly in standing but less so in walking; trunk muscles ERSP and GMED were strongly recruited in walking but less so in standing (Figure 1). Differences in muscle activity across standing and walking conditions were observed across perturbation directions and conditions (Figure 2). For example, agonist pairs of muscles such as MGAS/LGAS and PERO/TA which were activated similarly in perturbation responses during standing had distinct activation patterns in perturbation responses during walking.

**Figure 3:**
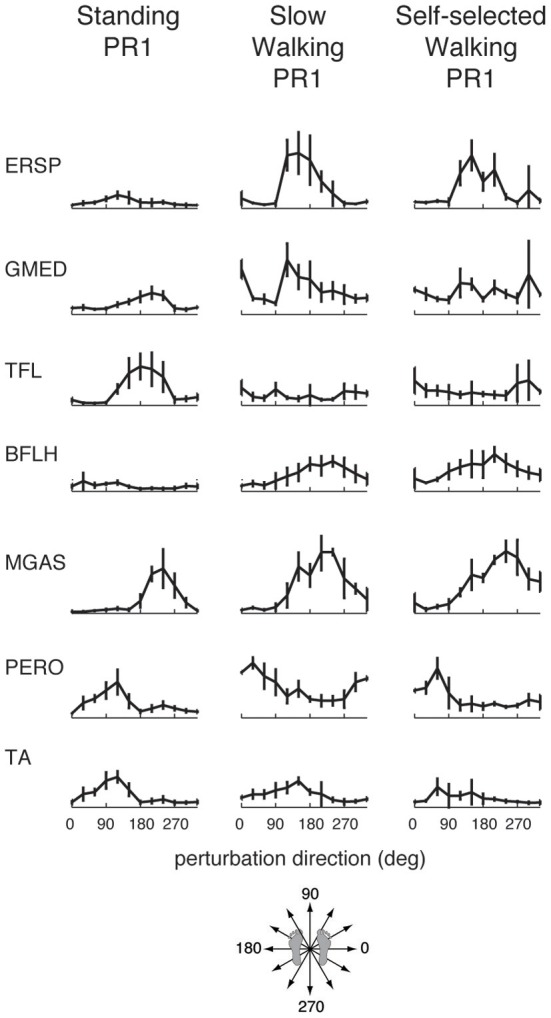
**Muscle tuning curves from perturbation responses during standing, slow walking, and self-selected walking speeds for a representative subject.** Shown are the mean tuning curves ± standard deviations in PR1 across trials in each perturbation direction. Some muscles have consistent tuning across perturbation conditions (standing, slow walking, self-selected walking), while other muscles have different tuning across conditions.

Muscle tuning curves revealed some differences in the directional tuning of muscles across perturbation conditions (Figure 3). For example, ERSP had the same preferred direction (forward/leftward perturbations) in all conditions, but was more highly activated in response to perturbations during walking compared to standing. TFL had a different preferred direction of activation in standing (forward/leftward perturbation) compared to walking (rightward perturbations). MGAS was recruited in similar directions (backward perturbations) and magnitudes in response to perturbations during both standing and walking.

### Common muscle synergies in perturbation responses during standing and walking

**Figure 4:**
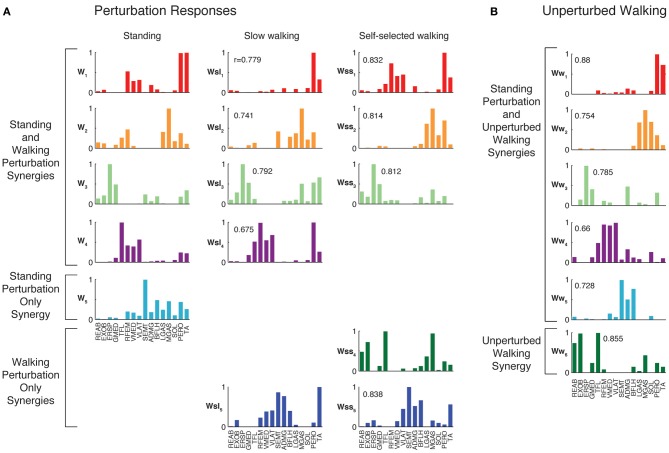
**Muscles synergies extracted from each experimental condition. (A)** Muscle synergies extracted from perturbation responses during standing, slow walking, and self-selected walking. All but one of the muscle synergies used in slow walking perturbation responses was similar to those used in standing balance postural responses. All but one of the muscle synergies used in self-selected walking perturbation responses was similar to those used in standing and/or slow walking postural responses. Correlations between each muscle synergy vector and the corresponding muscle synergy from standing balance are shown. **(B)** Muscle synergies extracted from unperturbed walking. Muscle synergies extracted from standing balance perturbation responses were similar to those extracted from the entire timecourse of many trials of unperturbed walking. In this subject, one additional muscle synergy was identified from unperturbed walking that was not identified in standing postural responses.

For each perturbation condition, on average, we identified 5.2 ± 0.9 muscle synergies (range 4–7, Figure 4) that explained the variance in muscle activation patterns across directions, time bins, and trials. In perturbation responses during standing, 5.6 ± 0.8 muscle synergies per subject (range 5–7) were sufficient to account for >90% total variability and >75% variability in each muscle and condition (all 3 time bins, 12 perturbation directions, across 5 trials of each) in the EMG data. In perturbation responses during slow walking, 4.9 ± 0.7 muscle synergies (range 4–6) were sufficient to explain the same amount of variability. In perturbation responses during self-selected walking, 5.1 ± 1.2 muscle synergies (range 4–7) were sufficient to explain the same amount of variability. Individual muscles were recruited by multiple muscle synergies; for example, PERO was recruited strongly by W_1_, but also was recruited by W_2_ and W_5_.

When muscle synergies were extracted separately from each perturbation condition, we found similarities in most of the muscle synergies identified (Figure 4). Of the muscle synergies extracted from perturbation responses during standing, 3.6 ± 1.0 (range 2–5) were identified in perturbation responses during slow walking, and 3.1 ± 0.9 (range 2–4) in perturbation responses during self-selected walking (Table 1). Of the 1 to 3 muscle synergies that were identified in perturbation responses to each walking condition that were not identified in perturbation responses to standing, one was similar between the perturbation responses during both walking conditions (e.g., Wsl_5_ and Wss_5_, Figure 4) for five subjects. The remaining muscle synergy identified in perturbation responses to self-selected walking was similar to a muscle synergy identified in unperturbed walking for most subjects, which will be discussed in detail later.

**Table 1:** **Number of muscle synergies used in each condition and number of similar muscle synergies used across different conditions**.

**Number of muscle synergies**	**Standing perturbation responses**	**Slow walking perturbation responses**	**Self-selected walking perturbation responses**	**Unperturbed walking**
Identified per condition	5.6 ± 0.8	4.9 ± 0.7	5.1 ± 1.2	6.9 ± 1.2
	(range 5–7)	(range 4–6)	(range 4–7)	(range 5–8)
Shared with standing perturbation responses	–	3.6 ± 1.0	3.1 ± 0.9	3.4 ± 1.1
		(range 2–5)	(range 2–4)	(range 2–5)

*The first row shows the total number of muscle synergies identified across all subjects for each condition. The second row indicates the number of muscle synergies identified in standing perturbation responses that were also identified in each of the other conditions*.

When muscle synergies extracted from perturbation responses during standing were used to reconstruct perturbation responses during walking, 1.7 ± 0.5 (range 1–2) additional muscle synergies specific to slow walking perturbations, and 2.4 ± 0.8 (range 1–3) additional muscle synergies specific to self-selected walking perturbations were required to explain the variability. Across subjects, the minimum VAF across muscles was significantly lower when only muscle synergies from standing perturbations were used to reconstruct walking perturbation responses than when muscle synergies extracted from walking perturbations were used (minimum VAFmus = 67.7 ± 11.6% vs. 82.4 ± 4.6%; *p* < 0.001). The reconstruction was improved once additional walking perturbation-specific muscle synergies were extracted, as evidenced by an increase in the minimum muscle VAF (minimum VAFmus = 82.3 ± 3.6%; *p* < 0.001). For all subjects, at least one of the walking perturbation-specific muscle synergies were similar to those identified from walking perturbations alone. For one subject all walking perturbation-specific muscle synergies were similar to walking perturbation synergies, for four subjects all but one were similar, for one subject all but two were similar, and in one subject all but three were similar.

Finally, we compared muscle synergies extracted above to those identified when all three perturbation conditions were combined. Across subjects, 6.6 ± 1.3 synergies could explain >90% VAF and >75% VAF in each muscle across all three perturbation conditions. For three subjects, all of the condition-specific muscle synergies were also identified from the combined data. For the other four subjects, at least one of the condition-specific muscle synergies were identified from combined perturbation response data. For muscle synergies that were similar across conditions, similar recruitment coefficients were identified from the combined dataset and each condition individually (VAF = 90.6 ± 3.6%, *r* = 0.90 ± 0.04).

### Muscle synergy tuning across perturbation conditions

**Figure 5:**
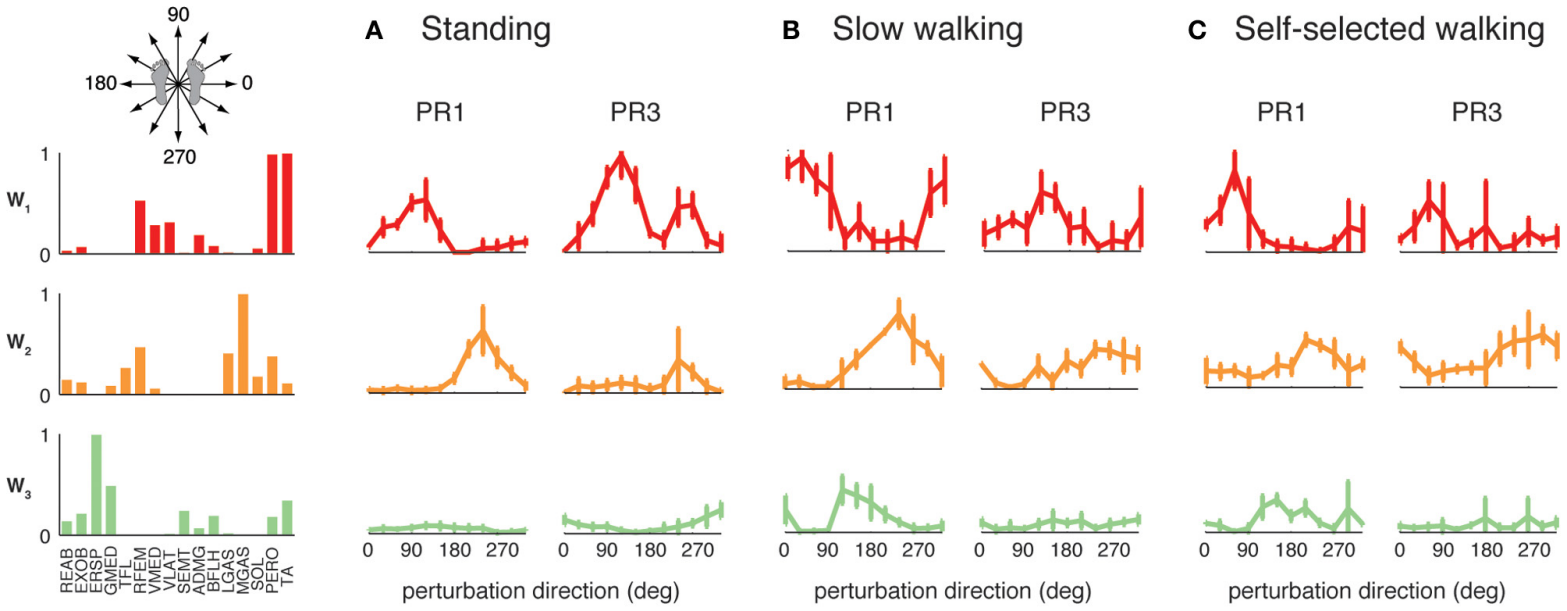
**Recruitment of muscle synergies common to perturbation responses during standing and walking.** W_1_, W_2_, and W_3_ were used in **(A)** standing perturbation responses as well as perturbation responses during **(B)** slow and **(C)** self-selected walking. W_2_ was recruited for backward perturbations in all conditions, whereas W_3_ was recruited for medial/lateral perturbations. W_1_ was recruited for anterior/posterior perturbations in standing perturbation responses, and for anterior and lateral perturbations in walking perturbation responses.

The differential recruitment of similar muscle synergies accounted for the differences in individual muscle patterns we observed during perturbation responses in both standing and walking. We found different magnitude and directional tuning of muscle synergies that were similar in standing and walking perturbation conditions (Figure 5). For example, W_1_, W_2_, and W_3_ were used in perturbation responses during standing as well as during both walking conditions. Muscle synergy recruitment tuning curves revealed differences in the both the magnitude and directional tuning of muscle synergies across perturbation conditions. For example, W_2_ was recruited for backward perturbations during both standing and walking, and W_3_ was recruited during postural responses to medial/lateral perturbations in all conditions, but both were more highly recruited in response to perturbations during walking compared to standing. W_1_ had a different preferred direction of activation in standing (forward and backward perturbations) compared to walking (rightward/forward perturbations).

**Figure 6:**
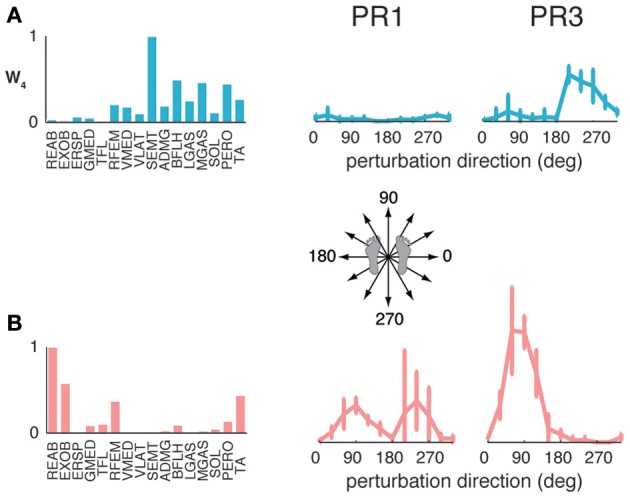
**Recruitment of muscle synergies identified in perturbation responses during standing but not walking.** Muscle synergies used in standing perturbation responses that were not used in walking perturbation responses were recruited for **(A)** backward or **(B)** forward perturbation directions, shown for two different subjects.

The muscle synergies identified in standing but not walking perturbation responses were generally recruited for forward or backward perturbations. Four subjects had a muscle synergy tuned for backward perturbations in standing but not walking perturbation responses (Figure 6A), and separate set of four subjects had a muscle synergy tuned for forward perturbations in standing that was not used in perturbation responses during walking (Figure 6B). For example, W_4_ was highly recruited during standing to move the CoM backward in PR3 during standing responses, but was not identified in perturbation responses during walking (Figure 6A), consistent with the goal of moving the CoM forward for forward progression. Similarly, we identified other muscle synergies that were highly recruited to move the center of mass forward in standing that were not used during walking (Figure 6B), presumably because the whole-body forward momentum carries the CoM forward during walking.

**Figure 7:**
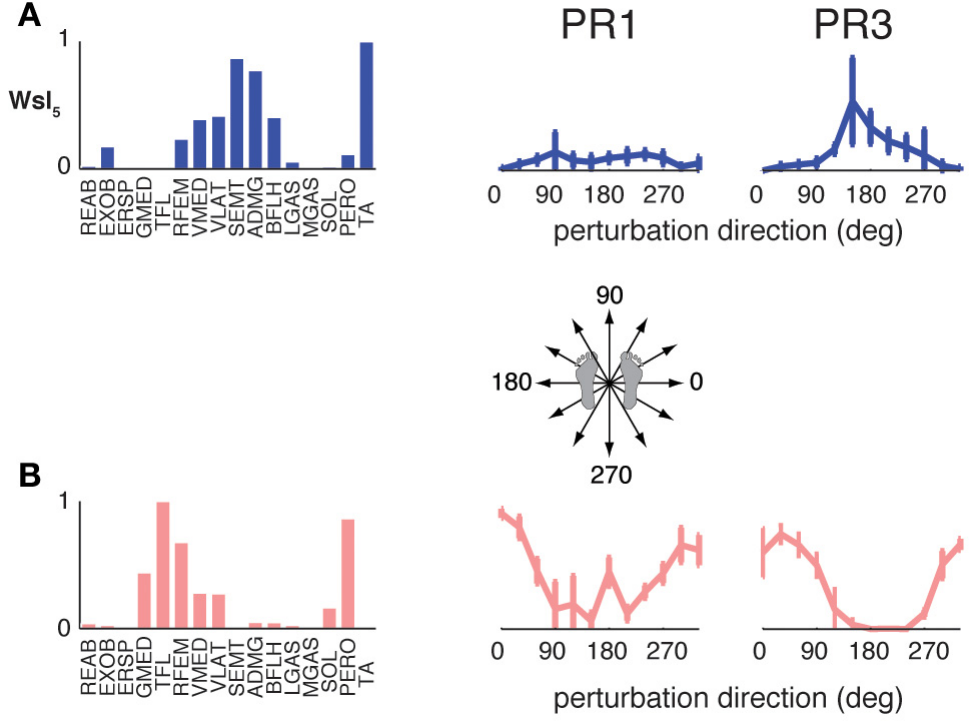
**Recruitment of muscle synergies identified in perturbation responses during walking but not standing.** Muscle synergies used in walking perturbation responses that were not used in standing perturbation responses were recruited for **(A)** leftward or **(B)** rightward perturbation directions, shown for two different subjects.

The muscle synergies specific to perturbation responses during walking were recruited for medial/lateral perturbations. For example, muscle synergies having strong contributions from hamstring muscles and TA (e.g., Figure 4, Wsl_5_ and Wss_5_) were recruited following leftward perturbations during walking (Figure 7A). Additional muscle synergies using PERO and TFL were recruited following rightward perturbations during walking (Figure 7B) and resembled a muscle synergy previously identified to emerges in postural responses during standing on one leg (Torres-Oviedo and Ting, 2010). An additional muscle synergy identified in perturbation responses during self-selected walking (Wss_4_), was similar to a muscle synergy identified in unperturbed walking (Ww_6_, Figure 4, *r* = 0.86), and was not strongly recruited in any perturbation direction, possibly playing a trunk stabilization role.

### Common muscle synergies in perturbation responses and unperturbed walking

Although unperturbed walking generally required a greater number of muscle synergies than perturbation responses during standing, the compositions of several muscle synergies used for walking and standing postural control were similar (see Figure 4). In unperturbed walking, on average, 6.9 ± 1.2 muscle synergies (range 5–8) from walking data from both walking speeds were sufficient to explain the variability in the data (VAF overall = 91.5 ± 1.2%, VAFmus = 91.0 ± 5.1%). Across subjects, 3.4 ± 1.1 of the muscle synergies used in perturbation responses during standing were similar to those used in unperturbed walking (range 2–5).

When standing perturbation muscle synergies were used to reconstruct unperturbed walking, and vice versa, reconstruction quality decreased (minimum VAFmus = 79.8 ± 2.7% decreased to 48.4 ± 13.6%, *p* = 0.001; and minimum VAFmus 87.4 ± 3.8% decreased to 56.2 ± 21.6%, *p* = 0.009, respectively). With the addition of condition-specific muscle synergies, reconstructions were improved (minimum VAFmus = 76.7 ± 7.3%, *p* = 0.002; and minimum VAFmus = 82.3 ± 4.6%, *p* = 0.007, respectively). When muscle synergies extracted from perturbation responses during standing were used to reconstruct unperturbed walking, 3.5 ± 0.9 (range 3–5) additional muscle synergies specific to walking were identified in order to meet reconstruction criteria, consistent with our observation that a greater number of muscle synergies are used in unperturbed walking as compared to perturbation responses during standing. When muscle synergies extracted from unperturbed walking were used to reconstruct perturbation responses during standing, 2.0 ± 0.8 (range 1–3) additional muscle synergies specific to standing perturbations were required to explain the variability.

Across subjects, 6.0 ± 0.6 synergies could explain >90% VAF and >75% VAF in each muscle when standing perturbation data and unperturbed walking data were combined. For all subjects, 5.1 ± 0.9 (range 4–6) of the muscle synergies identified from standing balance perturbations and 4.3 ± 1.0 (range 3–6) of the muscle synergies identified from unperturbed walking were also identified when standing and walking data were combined.

**Figure 8:**
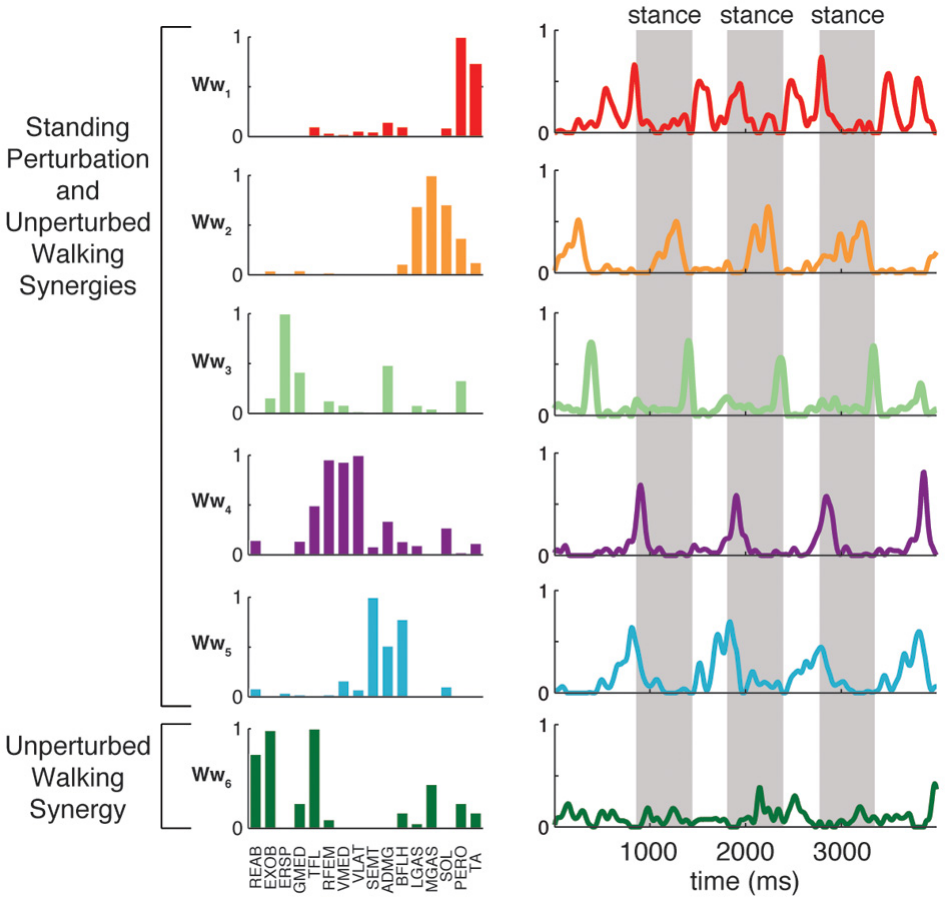
**Recruitment of muscle synergies identified in unperturbed walking.** Ww_6_ was identified from unperturbed walking but not during perturbation responses in any condition. Shown are the recruitment coefficients for a single trial of unperturbed walking at the self-selected speed. The gray boxes indicate stance phase.

Condition-specific muscle synergies reflected differences in the biomechanical demands of each conditions. For example, muscle synergies used in unperturbed walking that were not used in perturbation responses during standing were comprised of hip/trunk muscles and recruited throughout the gait cycle, suggesting they may play a role in trunk stabilization during walking (Figure 8, Ww_6_). The muscle synergies used in perturbation responses during standing but not in unperturbed walking either had large contributions from TFL and were active for medial/lateral perturbations, or had large contributions from TA and PERO and were active for anterior perturbations (not shown). Some muscle synergies used for posterior CoM movements were common to both perturbation responses during standing and unperturbed walking, but were not identified in perturbation responses during walking (i.e., W_4_, Figure 4).

## Discussion

Our results suggest that a common set of muscle synergies form a motor repertoire for both locomotion and reactive balance control. Our work unifies several different studies demonstrating a modular organization underlying variations in motor patterns across different walking and balance conditions. For example, step-by-step variation in muscle activity during walking as well as changes in muscle activity across walking speeds can all be explained by differential recruitment of walking muscle synergies as task demands change (Clark et al., 2010; Chvatal and Ting, 2012). Similarly, a common set of muscle synergies for reactive balance has been shown to underlie trial-by-trial differences in muscle activation patterns both within and across perturbation conditions (Torres-Oviedo and Ting, 2007, 2010; Chvatal et al., 2011). To bridge balance and walking behaviors, our study was the first to examine responses to perturbations during walking over 12 directions in the horizontal plane. We demonstrate that two different motor behaviors: walking and reactive balance, may in fact be constructed by a common set of muscle synergies. Our prior work also demonstrated that anticipatory changes in the walking pattern could also be described by change in the recruitment of a fixed set of walking muscle synergies (Chvatal and Ting, 2012). Thus, our study supports that idea that muscle synergies form a general repertoire of motor actions that can be recruited by a variety of different neural pathways for voluntary, rhythmic, and reactive motor behaviors (Chvatal and Ting, 2012).

Our findings support prior work demonstrating that changes in the modular organization of walking affect both walking and balance function. For example, the number of muscle synergies for walking is often reduced in the paretic limb of individuals with post-stroke hemiplegia (Clark et al., 2010) or their organization is modified (Gizzi et al., 2011). The reduction in muscle synergies is correlated not only to walking speed (Clark et al., 2010), but also to measures of balance control during standing (Bowden et al., 2010). In Parkinson's disease (PD), a reduced number of muscle synergies has been identified during walking (Rodriguez et al., 2013), but the relationship to postural instability, a cardinal sign of PD, is unknown. Further, degradation in upper limb function after stroke may be a result of changes in the modular organization of long-latency responses (Trumbower et al., 2010, 2013). These initial studies suggest that muscle synergy analysis may be a powerful tool for distinguishing specific deficits in muscle coordination leading to functional impairments that may be generalized across different motor behaviors.

Muscle synergies may therefore form the lowest level of the motor control hierarchy, recruited by parallel descending pathways mediating a wide variety of motor behaviors. Because muscle synergies only prescribe the spatial coordination of muscles to produce a motor function at a given instant in time, they may be concurrently recruited by different neural circuits mediating motor behaviors with common task-level goals. During locomotion, spinal mechanisms specifying the locomotor rhythm are known to be distinct from the spatial patterning of muscles across the limbs (Lafreniere-Roula and McCrea, 2005). Moreover, spatial patterning by muscle synergies is not modified by sensory feedback (Hart and Giszter, 2004; Cheung et al., 2005; Kargo et al., 2010) and is thought to be downstream of the rhythm generation mechanisms (Burke et al., 2001; McCrea and Rybak, 2008). Further evidence suggests that the same muscle synergies can also be recruited by cortical mechanisms to alter the locomotor pattern during motor planning and obstacle avoidance during locomotion (Drew, 1988; Drew et al., 2002, 2008), or anticipation of a balance perturbation during walking (Chvatal and Ting, 2012). Similarly, in reactive balance, the temporal patterning of the long-latency sensorimotor feedback response also appears to be independent of the precise spatial patterning of muscles defined by muscle synergies (Welch and Ting, 2009; Torres-Oviedo and Ting, 2010; Chvatal et al., 2011). Both voluntary and reactive balance responses are thought to be mediated by brainstem pathways (Schepens and Drew, 2004; Deliagina et al., 2008; Schepens et al., 2008; Lyalka et al., 2009) and may recruit different muscles synergies depending upon task demands. In reduced preparation, common muscle synergies are used in both automatic and reactive motor behaviors (Cheung et al., 2005; Kargo and Giszter, 2008; Roh et al., 2011). Therefore, muscle synergies may form a modular repertoire of actions that is specific to any given motor task, but recruited by a variety of neural pathways governing different motor behaviors.

As long-latency response to perturbations are modified with task-level goals during posture and movement, it is likely that common mechanisms govern reactive balance responses in standing and walking. Following perturbations during both standing (Horak and Nashner, 1986; Torres-Oviedo and Ting, 2007) and walking (Pijnappels et al., 2005; van Der Linden et al., 2007; Chvatal and Ting, 2012) muscles exhibit similar long-latency responses (~100 ms in humans). Moreover, long-latency responses coordinate the stance and swing limbs even when afferent inputs originate from a single limb (Dietz et al., 1989; Tang et al., 1998; Dietz and Duysens, 2000; Ting et al., 2000; Reisman et al., 2005; Duysens et al., 2013). Therefore, in contrast to short-latency responses that simply return the limb posture to the original configuration (Horak and Macpherson, 1996), long-latency motor patterns reflect abstract task-level goals such as controlling endpoint or CoM motion, which cannot be specified by independent joint-level controllers and require multisensory information. Moreover, it has been shown in both the upper and lower extremity that long-latency responses are modulated by motor planning, obstacle avoidance, and voluntary movement goals (Marsden et al., 1972, 1981; Carpenter et al., 1999; Pruszynski et al., 2009; Shemmell et al., 2010; Pruszynski and Scott, 2012) and are influenced by cortical contributions (Evarts and Tanji, 1976; Cheney and Fetz, 1984; Taube et al., 2006; Pruszynski et al., 2011). Accordingly, we previously found that reactive balance responses during whole-body reaching were modified to support target acquisition (Trivedi et al., 2010). Similarly, in this study, muscle synergy W_4_ is recruited in reactive balance during standing to move the CoM backwards, but not during walking, presumably so as not to inhibit forward progression. Likewise, our recent studies further demonstrate that the recruitment of muscle synergies during reactive balance reflect task-relevant error, e.g., deviation of the CoM from the upright condition (Safavynia and Ting, 2013), even when perturbations are imposed when the body is already deviated from the desired state. Therefore, long-latency mechanisms which restore the body to the upright equilibrium state during standing balance appear to be modified during voluntary movements and walking to support the return of the body to the desired trajectory (Pozzo et al., 1990; Borghese et al., 1996).

Here, differences observed in muscle synergies identified across walking and reactive balance conditions could be explained by differences in the task-level goals. Muscle synergies that appear to be specific to walking perturbations were similar to a previously identified muscle synergy in perturbations during standing on one leg (Torres-Oviedo and Ting, 2010). Therefore, these muscle synergies likely reflect the additional biomechanical challenges associated with single limb stance, whether it occurs during standing or walking. Although this muscle synergy was not used in unperturbed walking, it might be expected to contribute to walking conditions that require more non-sagittal plane control such as turning (Courtine and Schieppati, 2003). Further, muscle synergies used during sagittal plane motions in standing balance recovery were absent in perturbations to walking, consistent with the different goals for CoM motion in standing and walking. For example, some muscle synergies were recruited following forward perturbations in standing to move the CoM forward back to the original position, but were not identified in walking (Figure 6B); presumably the forward momentum of the body during walking was sufficient to move the CoM forward in response to forward perturbations during walking. Similarly, following backward perturbations which caused the body to fall forward, W_4_ was recruited in standing to move the CoM back to the original position (Figure 6A), whereas in walking these perturbations were consistent with the goal of moving the CoM forward for forward progression, so recruiting W_4_ was not necessary. Furthermore, these muscle synergies for posterior CoM movements identified in perturbation responses during standing but not walking were actually found to contribute to unperturbed walking, likely acting to decelerate the limb near the end of swing. Additionally, some muscle synergies involving hip and trunk muscles used in unperturbed walking that were not found in the standing perturbation responses measured here (Ww6, Figure 8) are similar to muscle synergies that emerge under more dynamic perturbations in standing balance that were hypothesized to stabilize trunk orientation (Safavynia and Ting, 2013). Together these findings suggest that muscle synergies provide a motor repertoire for the lower limbs and trunk across diverse balance and locomotor behaviors.

A complementary notion of modularity in locomotion has focused on the generation of temporal patterns during locomotion. A set of fixed temporal patterns governing muscle activity has been identified during walking, even when voluntary actions are concurrently performed (Ivanenko et al., 2004, 2005). These studies demonstrated that the neural mechanisms governing rhythmic generation of motor commands may also be modular. However, reactive balance is clearly a feedback response that depends closely on the characteristics of the perturbation (Lockhart and Ting, 2007; Welch and Ting, 2009), and as we have shown here, the temporal response to perturbations in reactive balance is likely controlled independent from the locomotor rhythm. When using NMF to identify fixed temporal patterns, the spatial muscle coordination pattern necessarily varies across timepoints, as detailed in Safavynia, 2012 (Safavynia and Ting, 2012). However, it is likely that the fixed temporal patterns for locomotor rhythm generation recruit spatially-fixed muscle activation patterns, such as the muscle synergies identified here. Dissociating modularity in the temporal and spatial domains requires a hierarchical analysis procedure to first identify modularity in spatial muscle activity, followed by modularity in temporal muscle activity (Safavynia and Ting, 2012, 2013). Our results suggest that reactive balance responses during walking are achieved by distinct mechanisms governing reactive balance, which may be superimposed upon the rhythmic walking patterns and recruit a common set of spatially-fixed muscle synergies.

Although muscle synergies can explain a large proportion of variability observed in muscle activity, using muscle synergy analysis to draw conclusions about neural mechanisms has limitations. First, the selection of the number muscle synergies could vary depending on the method used. Typically, to ensure that the results are physiologically interpretable, several different criteria must be achieved. Overall VAF is typically a poor indicator of the goodness of fit, whereas as local criteria, such as the VAF for a particular experimental condition or phase of gait reflect the degree to which actual muscle coordination patterns are reconstructed (Ting and Chvatal, 2010). Remaining variability in muscle activity may reflect sensorimotor noise, or other neural mechanisms such as short-latency reflex responses and may not be accounted for by recruitment of muscle synergies (Ting, 2007). In particular, heterogenic reflex responses (Nichols, 1994) may have different organization than muscle synergies for long-latency responses and voluntary movements (Trumbower et al., 2013). Further, motoneuron excitability may vary with joint angle (Hyngstrom et al., 2007), causing differences in apparent muscle synergy composition across postures. One strength of the muscle synergy analysis is that the number of independent motor command signals is not affected by crosstalk in EMG signals, however, crosstalk will alter the apparent composition of muscle synergies extracted. While it is not possible to dissociate co-activation from crosstalk in adjacent muscles, muscle synergy analysis can identify whether a muscle is activated independent from an adjacent muscle even in the presence of crosstalk. Here, we only performed comparisons of muscle synergies within the same subject, such that effects of any possible crosstalk would carry over from one condition to the next, and do not affect conclusions about similarity of muscle synergies across conditions. However, such crosstalk could be more problematic when comparing muscle synergies across subjects. Finally, the number of muscle synergies that can be identified is limited by the number of muscle signals analyzed as well as by the number of disparate conditions examined. Therefore, the number of muscles recorded and the number of experimental conditions must both be sufficiently high such that a sufficiently diverse set of muscle coordination patterns is represented in the dataset. Nonetheless, given the appropriate experimental design, muscle synergy analysis can help describe and potentially predict muscle coordination patterns in a functional and physiologically-relevant way.

A modular organization of spatial motor patterns may be a common principle for control of the upper and lower limbs useful for discerning mechanisms of motor deficit. Although muscle synergies for the upper and lower limbs may have different neural substrates, common principles likely govern their recruitment and organization. Muscle synergies for reaching may be organized by pyramidal cells in the motor cortex which project to multiple motoneurons. (Holdefer and Miller, 2002; Gentner and Classen, 2006; Overduin et al., 2008; Gentner et al., 2010; D'Avella et al., 2011). Pyramidal cells can also project to reticulospinal (Davidson et al., 2007) and propriospinal interneurons (Rathelot and Strick, 2009; Alstermark and Isa, 2012) which may explain residual motor function following stroke (Davidson and Buford, 2006). Muscle synergies for lower-limb movements are more likely encoded in the spinal cord (Hart and Giszter, 2004, 2010; Cheung et al., 2005; Kargo et al., 2010) and recruited by different neural pathways in the spinal cord, brainstem, and higher brain regions (Roh et al., 2011). By dissociating spatial from temporal aspects of motor coordination, muscle synergy analysis may aid in identifying neural impairments that are not evident in current clinical measures of motor function (Wolf et al., 1997; Coote et al., 2009; Hackney and Earhart, 2010). Such information may be important in identifying specific neural pathways that should be targeted for rehabilitation interventions, as well as for predicting generalized deficits in motor behaviors that are not specific to the particular tasks performed.

### Conflict of interest statement

The authors declare that the research was conducted in the absence of any commercial or financial relationships that could be construed as a potential conflict of interest.
